# Supplementary material for: Non-Canonical Male Meiosis in a Marine Gastropod, Littorina saxatilis
Source: Biology (Basel). 2025 Nov 9;14(11):1572. doi: 10.3390/biology14111572 (PMC12650740; doi:10.3390/biology14111572)
Supplement: Supplementary file 1 [file biology-14-01572-s001.zip › biology-3925065-supplementary.pdf]

Supplementary Materials

Suppl. 1. The Leptotene-to-Zygotene Transition in Male Meiosis of Littorina saxatilis *is Accompanied by a Bouquet-Like Stage, but not a Classical Bouquet*

Primary synapsis of homologous chromosomes occurs at the stage of late leptotene—early zygotene of mainstream meiosis [39]. This leads to the formation of the so-called "bouquet" due to the clustering of chromosome telomeres [6]. The primary synapsis zones are enriched with clumps of condensed chromatin, which is also visible in *L. saxatilis* spermatocyte nuclei stained with DAPI (Figure S1). At the same time, no pictures of the classical bouquet were observed, which suggests a possibly atypical course of the bouquet stage (see also Figure 11 in the main text).

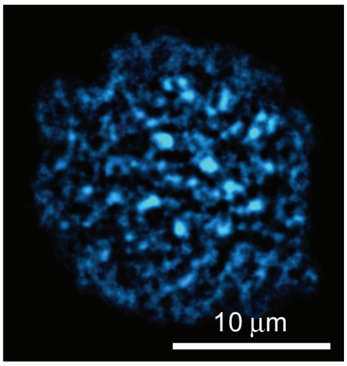

Figure S1. Beginning of homologous chromosome synapsis (the bouquet-like stage) in the spermatocyte nucleus of *Littorina saxatilis*. DAPI staining. Clusters of condensed chromatin are seen, which apparently reflects the onset of synapse.

Suppl. 2. Non-Canonical Littorina saxatilis *Male Meiosis, Including Karyosome Development*

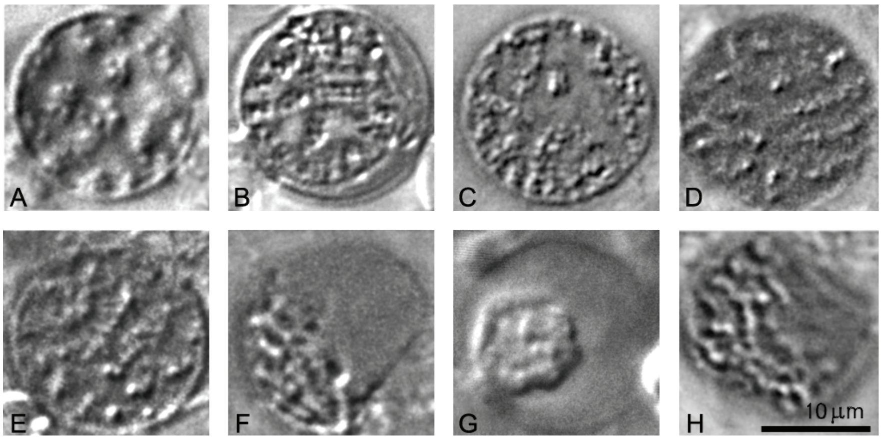

Figure S2. DIC imaging of unfixed primary spermatocytes and sequential atypical and non-canonical stages of meiotic prophase: zygotene (**A**), early pachytene (**B**), mid-late pachytene (**C**), diffuse stage (**D**), diplotene with "fuzzy" bivalents (**G**), pre-karyosome (**I**), complete karyosome (**F**), and post-karyosome (**G**) stages.
